# A Novel Method for Secondary Mandible Reconstruction to Re-Achieve a Native Condyle Position Comprising a New Design for Cutting Guides and New Positioning Devices

**DOI:** 10.3390/jpm14020181

**Published:** 2024-02-06

**Authors:** Fritjof Lentge, Philipp Jehn, Michael-Tobias Neuhaus, Stephan A. Bettag, Nils-Claudius Gellrich, Philippe Korn

**Affiliations:** Department of Oral and Maxillofacial Surgery, Hannover Medical School, Carl-Neuberg-Straße 1, 30625 Hannover, Germany

**Keywords:** free fibula flap, mandible reconstruction, CAD, patient specific

## Abstract

Secondary mandibular reconstruction using fibular free flaps (FFF) is a technical challenge for surgeons. Appropriate operation planning is crucial for postoperative quality control and is notably necessary for the (re-) achievement of a physiological condylar position, and the sensible expansion and shaping of the transplant. Computer-assisted planning may help to reconstruct mandibular defects in a patient-specific and precise manner. Herein, we present a newly-developed workflow for secondary mandibular reconstruction using FFF; it comprises digital planning and in-house manufacturing to perform precise secondary mandible reconstruction. This method utilizes a newly designed positioning device to ensure the precise positioning of the fibula segments in relation to each other and the mandibular stumps. The presented in-house-printed positioning device made it possible to achieve digital planning with high precision during surgery.

## 1. Introduction

For decades, the fibular free flap (FFF) has been considered the gold standard for osseous, and if necessary, the soft tissue reconstruction of large mandibular defects [1]. The long tubular bone represents an ideal “workpiece” for multisegment mandibular reconstruction, with acceptable donor-side morbidity [1]. Depending on the indication, FFFs may also be applied for primary or simultaneous reconstruction following ablative surgery, as well as for secondary (or tertiary) reconstruction in pre-existing defect situations.

Provided that the graft heals properly, the outcomes of osseous reconstruction can be measured from the physiological positioning of the condyles or the remaining mandibular segments, as well as the correct alignment of the fibula segments for subsequent dental rehabilitation, if desired. In cases of existing residual dentition, postoperative occlusion represents a clinically easily accessible control instrument for reconstruction quality [2,3]. Presently, computer-assisted design (CAD)-based surgical planning is widely used to improve the quality of mandibular reconstructions using FFF [4,5,6,7]. Furthermore, owing to the increased availability of affordable software solutions and three-dimensional (3D) printers, the in-house planning and manufacturing of cutting guides is increasingly being performed as a more cost-effective alternative to industrial surgical planning [7,8]. These techniques have resulted in a reduction in surgery and ischemia times, as well as lower deviations between surgical planning and intraoperative outcomes [4].

The simultaneous planning of primary mandibular resectioning and reconstruction is relatively simple as the condyles are usually in a physiological position during surgical planning. Furthermore, the existing dentition and contour of the mandible greatly facilitates the positioning of the fibula segments for subsequent dental reconstruction [2].

**Figure 1:**
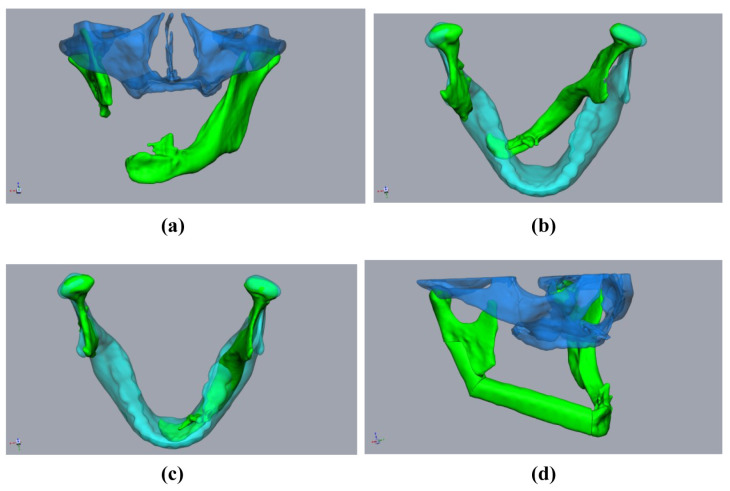
Planning using a “sample jaw”: A 59-year-old male patient underwent mandibular resection due to squamous cell carcinoma. The initial patient specific implant was lost due to exposition after radiation. (**a**,**b**) The defect situation with pronounced rotation of the mandibular stumps (green). Midface (clear blue). Sample jaw (clear cyan). (**c**) The mandibular stumps (green) aligned with the sample jaw (cyan). (**d**) Aligned and virtually resected mandibular stumps with fibula (green) and midface (clear blue).

**Figure 2:**
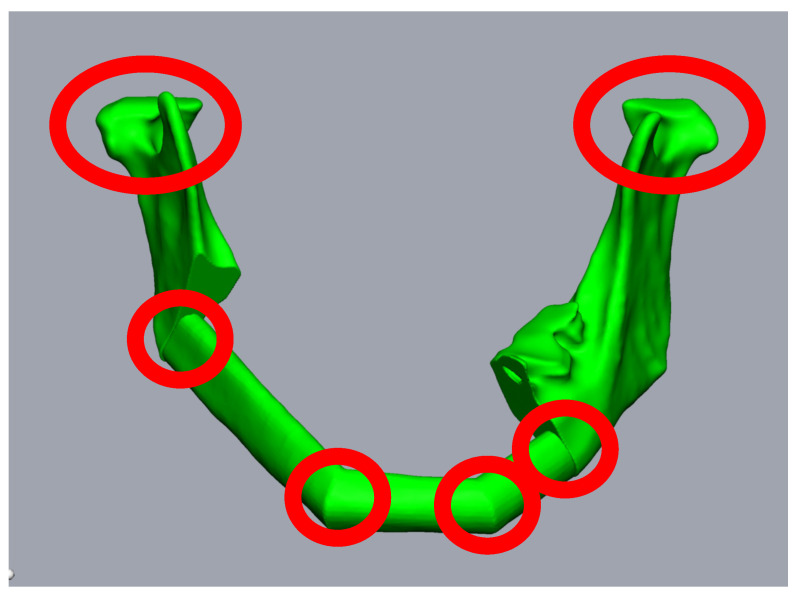
Planning of a three-piece-FFF. A 59-year-old female patient underwent mandibular resectioning and alloplastic reconstruction due to squamous cell carcinoma. Reconstruction using three FFF segments results in six points where intraoperative rotation errors can occur (red circles).

In contrast, secondary mandibular reconstructions place higher demands on surgical planning for FFF due to previous changes in the condylar position or the remaining mandibular segments. This is most obvious in segmental defects without alloplastic bridging, as the rotation of the remaining mandibular stumps is inevitable (Figure 1a,b). The same applies to cases with fractured or loosened alloplastic reconstructions, or osteolytic processes that have already led to the abolished continuity of the mandible. In addition to the correct repositioning of the remaining mandibular stumps in the planning phase, the correct positioning of the bony segments during surgery is complicated. Each contact point between the mandibular stumps and the FFF segments, or between the individual FFF segments, represents a predilection point for positioning errors, mainly rotational errors (Figure 2). Here, it is not only necessary to correctly position the bone grafts, but also the mandibular stumps, often against considerable soft tissue and scar resistance.

In this study, we present a new workflow using digital planning and in-house manufacturing to perform precise secondary mandible reconstruction. This method utilizes a newly designed positioning device to ensure the precise positioning of the fibula segments in relation to each other and the mandibular stumps.

## 2. Materials and Methods

### 2.1. Radiological Requirements

The acquisition of the 3D-datasets of both the complete mandible and the lower extremities is indispensable for digital planning. When imaging the mandible, high-resolution computed tomography (CT) (1 mm slice thickness) offers the best imaging quality; however, a high-quality Cone-Beam-CT (CB-CT) may alternatively be used. The scan field should include the entire maxillomandibular complex. If any previous CT scans are available, these images should be acquired during the preoperative and preplanned patient visits, before segmental mandibular resectioning.

Contrast-enhanced imaging of the head and neck region is not essential for surgical planning, although it may provide information regarding the vascular situation for subsequent anastomosis. However, color-coded duplex ultrasonography appears to be more appropriate for identifying potential recipient vessels.

To identify possible dental foci and teeth that are worthy of extraction, at least one current orthopantomogram, supplemented by single-tooth images if necessary, should be available at the time of surgical planning.

A high-resolution CT scan, with contrast-enhanced imaging of blood vessels, should also be available for the lower extremities. This imaging is necessary to ensure a three-vessel supply to the relevant lower leg before digital planning can take place based on CT.

### 2.2. Clinical Requirements

A clinical examination of the patient is obligatory. Attention should be paid to the quantity and quality of locoregional soft tissue. Several other factors should also be considered. First, the presence of intraoral or extraoral dehiscence or fistulas should be ascertained. Moreover, whether the soft tissues will remain true to the original repositioning of the mandibular stumps and the creation of an adequate recipient site should be ascertained. Furthermore, surgeons should assess whether the functional separation of the anatomical units (e.g., lower lip, neomandibular bone, and floor of the mouth) can be assumed even after the insertion of the osseous graft. Based on the results of these considerations, surgeons must decide whether the FFF must include a skin paddle. If so, it should be noted that this should be oriented either intraorally or extraorally.

Dental casts should also be created during the clinical examinations. This may be useful in the planning process to illustrate tooth-related issues and to create an occlusal splint.

Finally, the lower extremities should be examined for the presence of pathological changes, such as surgical scars, edema, or ulcerative skin lesions. Furthermore, the presence of physiological peripheral blood circulation, motor function, and sensitivity should be documented. Interpersonal preferences, such as stance leg and free leg, should be considered in surgical planning.

### 2.3. Technical Requirements

Software and hardware solutions must be available to enable segmentation of the bony structures (facial skull and fibula), 3D surgical planning, the digital creation of the cutting guides and positioning devices, and 3D printing. We used the Brainlab Elements software (Brainlab AG, Munich, Germany) to segment the bony structures, and subsequent digital surgical planning and construction of the cutting guides was performed with the program Geomagic^®^ Freeform Plus^®^ (3D Systems, Rock Hill, SC, USA), using a haptic input device developed by Geomagic^®^ TouchX^®^ (3D Systems, Rock Hill, SC, USA). Finally, 3D printing was performed using 3D printers developed by “Form 3B+” (Formlabs, Boston, MA, USA), using ”Surgical Guide”-resin (Formlabs, Boston, MA, USA).

### 2.4. Digital Operation Planning

**Figure 3:**
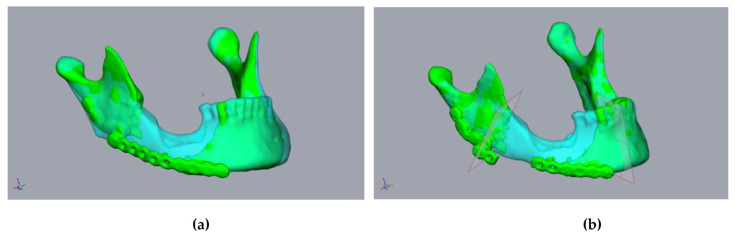
Planning using a CT scan before resectioning: A 55-year-old male patient presented with a fractured alloplastic reconstruction-plate after the partial resectioning of the mandibula due to radio osteonecrosis. (**a**) The mandibula before resectioning (clear cyan) and the current defect situation (green). (**b**) The repositioned mandibular stumps and planned resection planes.

Step (1) Import and align the segmented bones:

First, the segmented bone structures (mandible and fibula) were imported into the Freeform program and postprocessed. Non-relevant structures were removed, and the surfaces were smoothed while avoiding a loss of substance. To avoid loss of substance, first, artifacts were removed manually. A “buck layer” was then used to prevent the bony structures from being erased and automatic smoothing was performed. The models of the bony structures created in this way then served as the basis for constructing the cutting guides. Careful editing is important to ensure a good fit with the cutting guides. This was followed by the alignment of the mandibular segments. Special care must be taken, as this is a key point in surgical planning. There are two options for alignment. If it is possible to acquire a CT image of the original mandible without interrupting continuity in advance, this can be used in this step, which greatly facilitates the alignment of the mandibular stumps. We found that aligning the mandibular stumps with the correlated areas of the original mandible was sufficient (Figure 3a,b).

In cases where the 3D image of the original mandible is unavailable, the alignment of the mandibular stumps becomes more complicated. Therefore, we built an internal departmental database with artifact-free datasets of physiological mandibles, from which, a “sample jaw”, selected by age and sex, can be imported into the planning process (Figure 1b,c). The jaw could then be adjusted in terms of size, such that the condylar head of the temporomandibular joint is aligned. Any modifications to the anteroposterior dimensions of the model jaw could also be performed. This is made possible by the fact that modern planning programs—such as Freeform+^®^ used here—allow both a proportion-preserving enlargement or a reduction of the digital model jaw, as well as adjusted stretching or compression. This enables the key points of the selected sample jaw to be precisely adjusted. The condyles, jaw angles, mentum, and mandibular midline, as well as the dental arch, were considered as key points to adjust. If the patient’s midface was segmented in advance, the size-adapted mandible was then closed to such an extent (i.e., rotated along the axis of the temporomandibular joints) that the bimaxillary complex could be assessed. When the alignment of the model jaw was complete, the alignment of the mandibular stumps and planning of the FFF was performed analogously using the above procedure (Figure 1).

Step (2) Definition of the resectioning planes:

The mandibular stumps to be resected were subsequently determined, and the resectioning planes were inserted into the plan accordingly (Figure 3). Owing to the general inaccuracy of the segmentation, the resectioning a few millimeters of bone was performed, even on radiologically inconspicuous jaw stumps. This achieved a defined contact between the mandible, the graft, and the fresh osseous wound surface which is required for the optimal healing of the graft. At this point, it should already be noted that the resectioning planes diverge caudally so that the fibula graft can be inserted later without any constraints.

**Figure 4:**
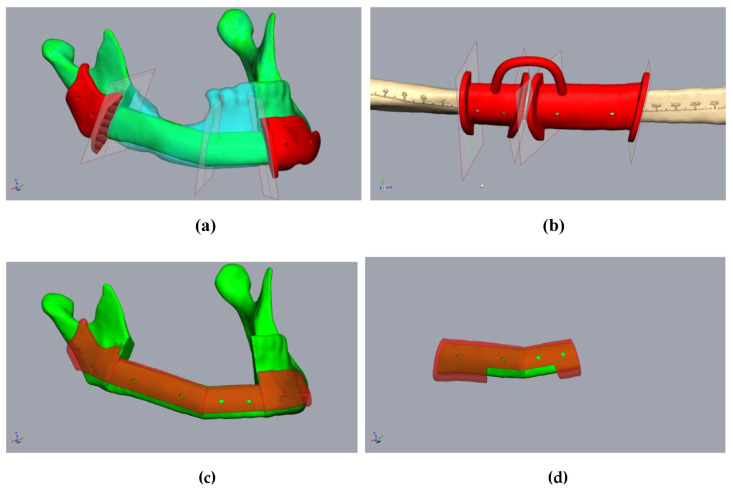
Planning of the resection-guides, cutting-guides, and positioning device in the same patient as in Figure 2. (**a**) The mandibula with positioned fibula segments (green), considering the original mandible (clear cyan), osteotomy lines, and cutting guides (red). (**b**) Fibula (beige) with osteotomy lines (transferred from Figure 3a) and cutting guide (red). (**c**) The mandibula with fibula segments (green) and positioning device at the mandibula (clear red). (**d**) Fibula segments (green) with the positioning device to fix the fibula before removal (clear red).

**Figure 5:**
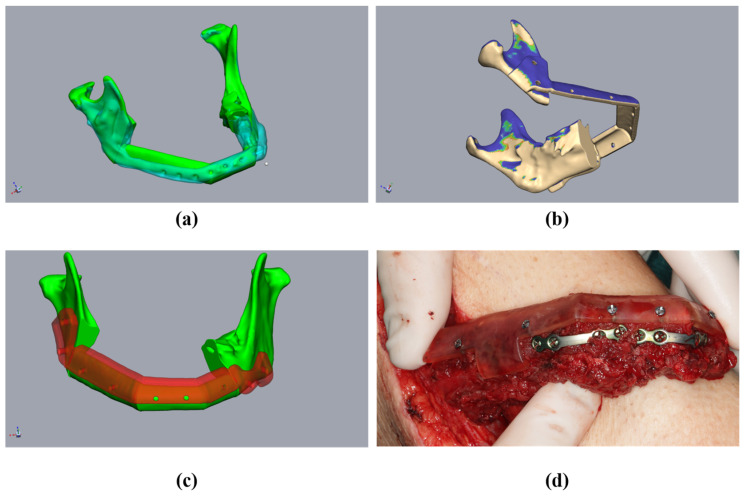
Planning of a three-piece-FFF. A 59 year-old female patient underwent mandibular resectioning and alloplastic reconstruction due to squamous cell carcinoma. (**a**) The mandibula with positioned fibula segments (green) considered the original mandible (clear cyan). The actual situation with alloplastic reconstruction (clear cyan). (**b**) Checking the mandibular and positioning device for undercuts. Blue means undercut. (**c**) The mandibula with fibula segments (green) and positioning device at the mandibula (clear red). (**d**) The osteotomized fibula with the positioning device and performed osteosynthesis before the onset of ischemia time.

Step (3) Planning of the fibula segments and creation of the cutting and positioning guides:

The single fibula segments were positioned and the resultant cutting planes were transferred to the fibula. The cutting guides for the fibula and mandibular stumps were constructed (Figure 4a,b). An offset of 1 mm in the fibula region to account for the soft tissue cuff, and an offset of 0.3 mm for the mandible, were found to be effective in ensuring the optimal intraoperative fitting of the guides. The design of the cutting guides should provide a clear shape fitting. To ensure this, prominent anatomical structures should be enclosed by the cutting guides. Encircling the mandibular margin with small “feet” is also helpful to ensure exact intraoperative positioning. At least two drill holes were implemented per fibular segment and per mandibular stump for the intraoperative fixation of the guides. The vectors of the drill holes were subsequently transferred to the digital fibular segments in situ. It must be noted that at this stage, the “drill holes” for fixing the cutting guides did not interfere with the osteosynthesis, which may be subsequently performed on the lower edge of the mandible or the transplanted fibula. The positioning device was then constructed based on the positioned mandibular stumps and fibular segments (Figure 4c,d). The positioning device considered the fibula segments and positioned the mandibular stumps during operation planning (Figure 4c). The design of the positioning device differs significantly from the creation of conventional cutting guides in some respects. The existing vectors of the drill holes, of both the cutting guides and the transplanted fibula segments, ensured secure positioning. This eliminated the need for complex form fitting, which is required for cutting guides, and it reduced the contact surfaces on the bone. This simplified both the design and the intraoperative positioning of the device. During the design of the mandibular components of the positioning device, it should be taken into account that the simultaneous fixation of both mandibular segments was required. However, due to the mobility of the mandibular stumps, it is generally not necessary to determine a common insertion direction. When the mandibular segments were designed, the design of the insertion area for the fibula segments was carried out. It should be noted that the fibula segments were already fixed together before transposition into the recipient area. Accordingly, it is important to ensure that the graft can be inserted in a caudal direction without undercuts (Figure 5b). A heat map to visualize any undercuts is helpful here. The layout of the fibula component in the positioning device can now be determined in such a way that the graft can be inserted and fixed without any problems by reusing the drill holes of the fibula cutting guide. There must also be sufficient cutouts on the caudal edge of the positioning device for osteosynthesis to be performed (Figure 4c,d and Figure 5c). It may be necessary to realign the resectioning planes on the lower jaw or the drill holes of the cutting guides. This ensures the safe positioning of the fibula intraoperatively in the resected mandible. The same drill holes were then used to attach the device based on the vectors of the drill holes of the cutting guides. In this sense, the secure positioning of the device on the mandible, and the fibula segments on the device, can be performed in accordance with the osteotomies performed. This is because the drill holes of the fixation screws are reused. When designing a positioning device, it is important to ensure that areas concerning at least one osteosynthesis per osteotomy are excluded. Furthermore, undercuts must be avoided to ensure that the fibula can be inserted intraoperatively. According to the cutting guides, an offset of 1 mm should be maintained in the area of the fibula graft, and 0.3 mm should be maintained in the area of the mandibular stumps.

The additional creation of anatomical models (e.g., of the resected jaw or a “dummy” of the fibula graft) can be helpful in ensuring the intraoperative orientation and control of individual intermediate steps.

**Figure 6:**
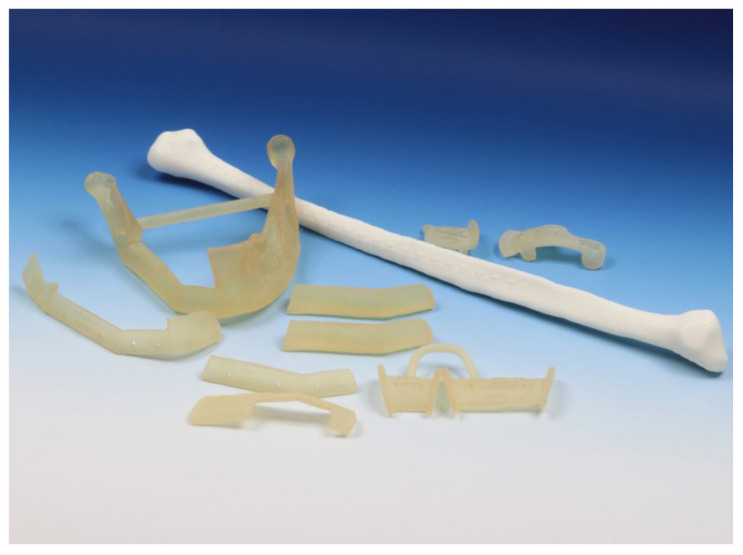
Cutting guides and anatomical models before sterilization.

The guides and models created were then printed (“Form 3B+”-Printer and ”Surgical Guide”-resin (both Formlabs, Boston, MA, USA)) for sterilization (Figure 6).

### 2.5. Implementation of the Surgery

**Figure 7:**
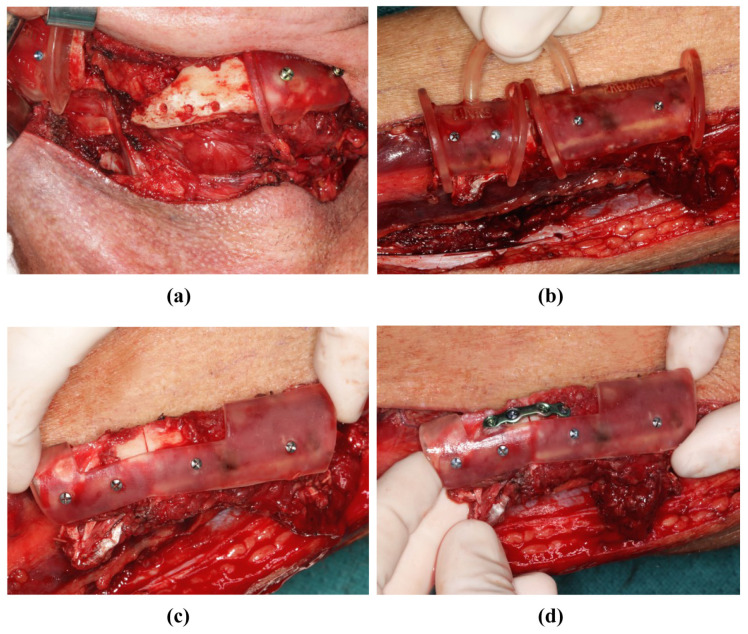
Mandibular resectioning and harvesting of the fibula (same patient as in Figure 2). (**a**) The mandibula with attached cutting guides. (**b**) The fibula with attached cutting guide. (**c**) The osteotomized fibula with attached positioning device before the onset of ischemia time. (**d**) The osteotomized fibula with positioning device, and was osteosynthesis performed before the onset of ischemia time.

**Figure 8:**
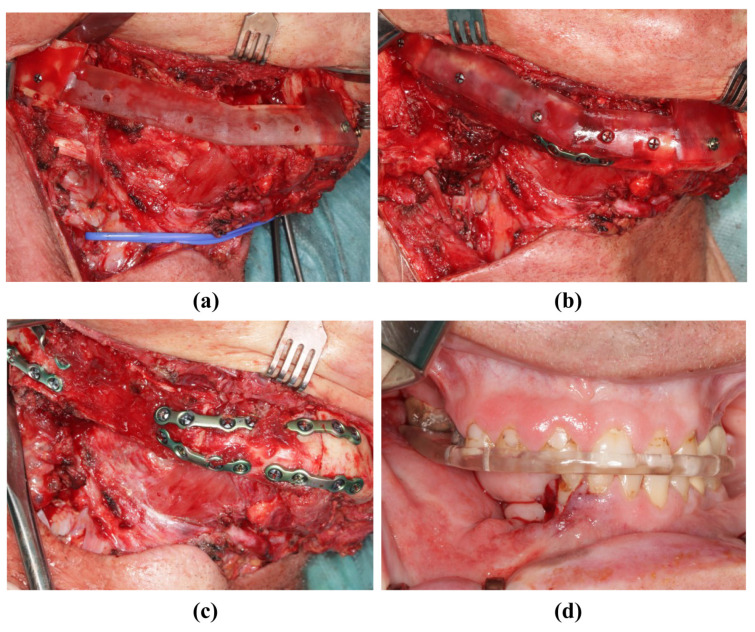
Insertion of the transplant into the mandibula (same patient as in Figure 2). (**a**) The resected mandible with positioning device. (**b**) The fibula fixed to the positioning device. (**c**) The fibula without the positioning device and completed osteosynthesis. (**d**) Postoperative occlusion with vavor.

During the surgical execution of preoperative planning, care must be taken to ensure the meticulous positioning of the cutting guides (Figure 5a,b), as incorrect positioning is the primary source of inaccuracy. In particular, cutting guides on the mandible must be precisely aligned because their position determines the final configuration of the mandible. Inaccuracies that arise here can only be compensated later by retaining the original surgical plan. Conversely, the positioning of the cutting guide on the fibula may be more robust against minor positional deviations; by temporarily fixing the segments to the guide with screws, these segments can be accurately repositioned later in the positioning guide, even if the fit is not optimal (Figure 7a,b). After the development of the FFF technique, the positioning guide can be used to perform osteosynthesis of the fibular segments, even before the pedicle is cut (Figure 5d and Figure 7c). The positioning device can also be attached to the mandible before the onset of ischemia (Figure 8a). The graft can then be fixed to the positioning device for the duration of the anastomosis (Figure 8b). After the anastomoses were performed, at least one osteosynthesis was performed between the FFF and the mandible. After removing the device, all the remaining osteosyntheses were performed (Figure 8c). If created in advance, a dental splint could be inserted for verification and to support the surgical results (Figure 8d).

## 3. Discussion

The FFF has been successfully used in mandibular reconstruction for decades [1]. Therefore, current developments mainly consider methods which will ensure that osteotomies will be performed more precisely, and that the graft will be inserted into the recipient area more precisely [3,4,9,10,11].

Initially, the configuration of the graft that was harvested, and thus, the angulation and positioning of the FFF in the recipient site, were performed intraoperatively, and they were based on the surgeon’s operative experience. More recently, CAD-based methods for surgical planning and the determination of the graft design in particular have been widely described [3,4,6,7,8,11]. Both industrial surgical planning (concerning the production of the cutting guides to be applied intraoperatively) and so-called “in-house” planning, are common procedures. However, due to the wide availability of reliable planning software and 3D printers, in-house planning is becoming increasingly popular [3,4,6,7,8,11], partly due to the significant advantage of shorter communication paths [12].

Prior studies have shown that digital planning and the preoperative fabrication of cutting guides and resectioning templates significantly simplifies the surgical performance of FFFs [13]. Both the total operative time and ischemia time can be reduced using CAD planning [2,3]. Simultaneously, CAD planning allows for better surgical outcomes in terms of graft expansion, configuration, and positioning [13].

In the surgical planning of secondary mandibular reconstruction, the positioning of the mandibular stumps in the planning stage, as well as in the precise implementation of surgical planning, are essential steps. Maintaining the physiological condylar position in three dimensions is also important to ensure the unrestricted postoperative articulation of the mandible [2]. Furthermore, the transplanted bone should be configured in such a way that the extraoral contour of the lower face is preserved as far as possible, or so that it appears harmonious, while the osseous reconstruction of the mandible is used as the basis for dental rehabilitation. Accordingly, the positioning of the grafted bone should allow for the prosthetically reasonable positioning of the dental implants [13].

Planning for simultaneous reconstructions in cases of mandibular continuity resectioning is simple, as the condyles are usually physiologically positioned. Positioning the fibula is also much easier because a residual dentition, or at least the contour of the original mandible, can usually be used for orientation. Before secondary reconstruction, there are often aggravating circumstances, the most serious of which appears to be an initial situation with free-ending mandibular stumps, without temporary alloplastic reconstruction. In such cases, a varying degree of rotation in the remaining mandibular segments is generally seen. The same applies to fractured osteosynthetic materials or osteolytic processes that have already led to a pathological fracture. However, a reasonably pronounced change in the condylar position can also occur in cases of alloplastic reconstructions that are performed in advance [14].

Mispositioning the inserted grafts and the remaining mandibular stumps is a common and well-known pitfall in both autologous and alloplastic mandibular reconstructions [14,15,16,17]. Since then, numerous procedures have been published to increase intraoperative precision while shortening the duration of surgery to the greatest extent possible; patient-specific planned solutions are commonly used in this context. For alloplastic implants, a functionalized design is used to shape-fit implants for unambiguous positioning [16]. For autologous reconstructions, such as FFF, CAD-based, patient-specific, planned cutting guides are the most commonly used methods to achieve a good fit of the bony segments [4,6,7]. Nevertheless, the fibular segments and mandibular stumps form a chain of bodies that can be freely positioned relative to each other, similar to a beaded necklace. Positioning devices have previously been reported to allow the correct alignment of segments [10,17]. Following the principles previously proposed by Wang et al., the use of the fixation holes of the cutting guide for subsequent implant positioning was shown to be promising. However, Wang et al. did not elaborate on their technical approach [10]. A similar effect can be achieved using patient-specific implants, which allow for the positioning of the mandibular stumps and the fitting of the fibular segments to the plate [18]. However, this procedure requires the external fabrication of the implants and a relatively large amount of osteosynthesis directly onto the graft. The intraoperative use of navigation systems has been reported as another possibility for aligning FFF segments, but this requires considerable intraoperative effort [19].

The proposed planning and surgical procedure for secondary mandibular reconstruction, using FFF represents an extension of the classic digitally planned FFF. The core aspects include the achievement of a physiological mandibular configuration and a condylar position that corresponds as closely as possible with the original. By transferring the fixation holes for the fibula from the cutting guide to the positioning device, a reliably reproducible FFF position in the recipient region is possible. Furthermore, the positioning device simplifies the temporary fixation of the graft, thereby helping to shorten the ischemia time. By fixing the FFF-graft to the recipient area in a more simplistic manner, the positioning device enables the precise and simple osteosynthesis of all bony parts. The three-dimensionally complex manual positioning and holding of the FFF segments is completely performed using the positioning device. If such an operation is performed via a two-team approach, the procedure presented here can be used to fix the fibula segments to each other before the onset of the ischemia time at the same time that the recipient region is being prepared; this can further reduce the duration of the operation depending on the overall surgical situation. If the entire treatment procedure is considered, the presented procedure leads to a reduction in operation-time but to a extension in time spent on surgery planning. However, the additional time required for surgical planning is limited to approx. 60–90 min, depending on the complexity of the case, provided that the planning is carried out by an experienced surgeon. In our experience, the use of radiological datasets generated before continuous resectioning is the most accurate way to plan the operation. When superimposed on the current situation, this allows the original condylar position and actual extent of the defect to be determined accurately on a patient-specific basis. Alternatively, the alignment of the mandibular stumps can be performed on a “sample jaw”. Modern CAD planning programs offer numerous options for adjusting the dimensions and positioning of a “sample jaw”. Nevertheless, the use of a “sample jaw” is primarily an orientation aid for positioning the existing jaw stumps and placing the FFF segments. In particular, inlaying osteosynthetic material significantly complicates the correct segmentation of mandibular stumps and often results in a poor fit of the cutting guides.

The presented technique was shown to be highly effective, but there are some limitations which should be considered. Although a preoperative splint can be created and inserted intraoperatively to secure occlusion, the procedure presented herein is not occlusion-based. Thus, especially in cases of long-standing defect-related malocclusion, some deviations in the occlusion may occur even if surgical planning is implemented correctly. As FFF is usually prepared for prosthetic dental rehabilitation, this limiting factor of the procedure can usually be remedied by providing additional patient care [20]. Edentulous patients require increased demands regarding the correct positioning of the FFF segments. Regardless of the planning and surgical procedure used, there is no possibility of occlusion-based intraoperative positioning of the mandibular stumps occurring using a splint. However, since the procedure presented herein is based on the bony anatomy of the mandible, surgical planning can also be carried out without restriction in edentulous patients, as illustrated above. The intraoperative use of the purely bone-anchored positioning device can also be carried out, regardless of the patient’s dentition. Therefore, careful positioning of the cutting guide in the mandible is essential. If the deviations are acceptable, it is still possible to insert the graft using the positioning device. However, the resultant deviation remains unchanged in the results of the operation. Accordingly, cutting guides must be designed to guarantee unambiguous positioning.

## Data Availability

Data are contained within the article.
